# Conceptual Model of Comprehensive Research Metrics for Improved Human Health and Environment

**DOI:** 10.1289/ehp.10925

**Published:** 2008-02-12

**Authors:** Jill A. Engel-Cox, Bennett Van Houten, Jerry Phelps, Shyanika W. Rose

**Affiliations:** 1 Battelle Memorial Institute, Arlington, Virginia, USA; 2 Division of Extramural Research and Training, National Institute of Environmental Health Sciences, National Institutes of Health, Department of Health and Human Services, Research Triangle Park, North Carolina, USA; 3 Battelle Memorial Institute, Durham, North Carolina, USA

**Keywords:** conceptual model development, environmental health research, metrics development, performance measurement, research impact evaluation

## Abstract

**Objective:**

Federal, state, and private research agencies and organizations have faced increasing administrative and public demand for performance measurement. Historically, performance measurement predominantly consisted of near-term outputs measured through bibliometrics. The recent focus is on accountability for investment based on long-term outcomes. Developing measurable outcome-based metrics for research programs has been particularly challenging, because of difficulty linking research results to spatially and temporally distant outcomes. Our objective in this review is to build a logic model and associated metrics through which to measure the contribution of environmental health research programs to improvements in human health, the environment, and the economy.

**Data sources:**

We used expert input and literature research on research impact assessment.

**Data extraction:**

With these sources, we developed a logic model that defines the components and linkages between extramural environmental health research grant programs and the outputs and outcomes related to health and social welfare, environmental quality and sustainability, economics, and quality of life.

**Data synthesis:**

The logic model focuses on the environmental health research portfolio of the National Institute of Environmental Health Sciences (NIEHS) Division of Extramural Research and Training. The model delineates pathways for contributions by five types of institutional partners in the research process: NIEHS, other government (federal, state, and local) agencies, grantee institutions, business and industry, and community partners.

**Conclusions:**

The model is being applied to specific NIEHS research applications and the broader research community. We briefly discuss two examples and discuss the strengths and limits of outcome-based evaluation of research programs.

The mission of the National Institute of Environmental Health Sciences (NIEHS) is to reduce the burden of human illness and dysfunction from environmental causes. This mission is furthered partly through funding of extramural research in science that focuses on the cellular and molecular basis of environmentally induced disease. Other types of projects funded as part of the extramural research portfolio include epidemiologic and community-based participatory research, as well as worker training and education. NIEHS is achieving its mission by focusing on diseases for which there is a strong indication of an environmental component, and for which there is high or increasing prevalence in the U.S. population (e.g., asthma); by fostering integrated research teams testing complex hypotheses that address the interplay of environmental and other factors, such as genetics, sex or gender, age, and lifestyle; and by developing initiatives identifying the complex factors in the environment that can increase the risk of disease by supporting basic research that develops the scientific basis for health decisions, as well as applied research that fills gaps in understanding of environmental health risks (NIEHS 2006b).

Given the complexity and diversity of research, program evaluation is critical to understanding and documenting the effectiveness of funded research in illuminating the linkages between the environment and human health. Mandates such as the Government Performance and Results Act of 1993 have required research agencies to look beyond measures of output (e.g., publications produced) toward metrics related to long-term outcomes on public health. Guidance from the U.S. Office of Management and Budget Program Assessment Rating Tool (PART) requires that outcomes of a program (managed by a single entity) be linked to a clear set of program and agency goals, yet be external to the research program (Office of Management and Budget 2006). When reviewing fundamental research programs using the PART guidance, managers of these programs face significant challenges in demonstrating a link between traditional research outputs and outcomes (Cozzens 1997). Health and environmental research organizations such as NIEHS have been challenged to define and measure outcomes distant in time and space from environmental health research (Van Houten et al. 2000). Outcome-based measures of accountability for research grants are inherently difficult, because by definition in the Federal Grants and Cooperative Agreement Act of 1977, grants have indirect benefit to and little substantial involvement by federal agencies.

The objective of this study was to develop a conceptual framework to measure the impact of environmental health research programs on human health, the environment, and the economy, even when the impact may be indirect or diffuse.

## Approach

Describing a research portfolio as comprehensive and multidisciplinary as that of NIEHS and measuring its effect on environmental health require a strategic approach that acknowledges all of the potential components of the research process and the application of that research to society in order to ultimately improve human health and quality of life. To design this approach, we developed a comprehensive logic model describing the agency’s extramural research portfolio from grant award through ultimate outcomes. Logic models are graphic depictions of the relationship between a program’s activities and its intended outcomes (Centers for Disease Control and Prevention 2005; Department of Health and Human Services 2002) and help to explain a program’s “theory” or the underlying structure of how the program is intended to work (Chen 2005). Besides being an evaluation tool, a logic model can also help program managers describe, and make explicit, how program “performance” is designed to achieve outcomes (McLaughlin and Jordan 1999). Research programs have extended traditional program logic to illustrate how research contributes to topics that inform federal decisions about protective health standards (e.g., National Research Council 2004).

To broaden this conception and to incorporate requirements for outcome-based program evaluation, our logic model of a research program provides a visual and conceptual representation of what broad impacts the research program is likely to have and how the impacts are achieved. The simplest structure that defines the impact of research on society is a linear progression:


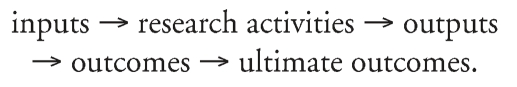


We chose this format because much of the theoretical and methodological literature describing the research process either explicitly or implicitly provides information on the inputs, activities, outputs, or outcomes of research, as well as describing how these elements of the research process can be linked to one another (Powers et al. 2006). Even though the process may not be linear, our focus is on the influence of specific research program inputs on a range of outcomes and does not attempt to evaluate all the influences of a particular outcome. Definitions of the logic model components are presented below.

Inputs are resources that feed into the research program from NIEHS, other federal agencies, research institutions, and community and business partners (e.g., funding, staff qualifications, technical assistance, grantees, organizational resources, community resources).

Activities are actions that describe how the inputs are used to carry out the research program or project (e.g., grant awarding, exposure/risk assessments).

Outputs are the direct products of the research activities, such as publications, presentations, and new funding applications, as well as patents and products.

Outcomes are benefits or changes resulting from the use of the research outputs. Outcomes are defined further as short term, intermediate, long term, and ultimate. Assigning time frames to the four levels of outcomes is difficult, because the length of time taken is highly variable depending on the individual outcome and the many factors that may affect it. Short- to long-term outcomes may include:

- Translation into or adoption of policy or administrative decisions, clinical guidelines, improved allocation of resources, setting of health targets, development of criteria for evaluative and inspective bodies, commercial development and availability of products, behavioral change among practitioners, and the use of commercial products (Buxton and Hanney 1996; Hanney et al. 2003)
- New and improved products and processes; methods of organizing, managing, and evaluating products and environments; improved safety of products and work environments; and individual and sector productivity rates (Bozeman 2003)
- The incidence, magnitude, and duration of social change (Bozeman 2003).

Ultimate outcomes of environmental health research may include:

- Health and social welfare gain and national economic benefit from commercial exploitation and a healthy workforce (Buxton and Hanney 1996; Hanney et al. 2003)
- Environmental quality and sustainability, improved health care and healthy longevity, and provision of basic needs to the population (Bozeman 2003)
- International balance of trade (i.e., the relation of exports to imports of various countries), energy independence, gross national product, and quality of life (Rubenstein and Giesler 1988).

Two other components of the logic model as they related to the NIEHS extramural research portfolio include contextual factors and reservoir of knowledge. Contextual factors could potentially affect the research environment through availability of resources or shifts in research or policy priorities that create constraints or opportunities for the research program. Examples include political or society interests, external triggers such as a disease outbreak, state of the economy, and other national and global socioeconomic influences.

Reservoir of knowledge represents the accumulation of understanding, knowledge, and previous research that may or may not be directly related to the NIEHS extramural research portfolio but contributes to the development of and, in turn, is contributed to as a result of the research activities described within the model. This “knowledge pool” is difficult to measure concretely, but encompasses both research and the interaction of individuals that “interact and produce innovation and discovery through unpredictable paths and at uneven intervals” (Cozzens 1997).

## Conceptual Logic Model and Submodels for Research Metrics

**Figure 1:**
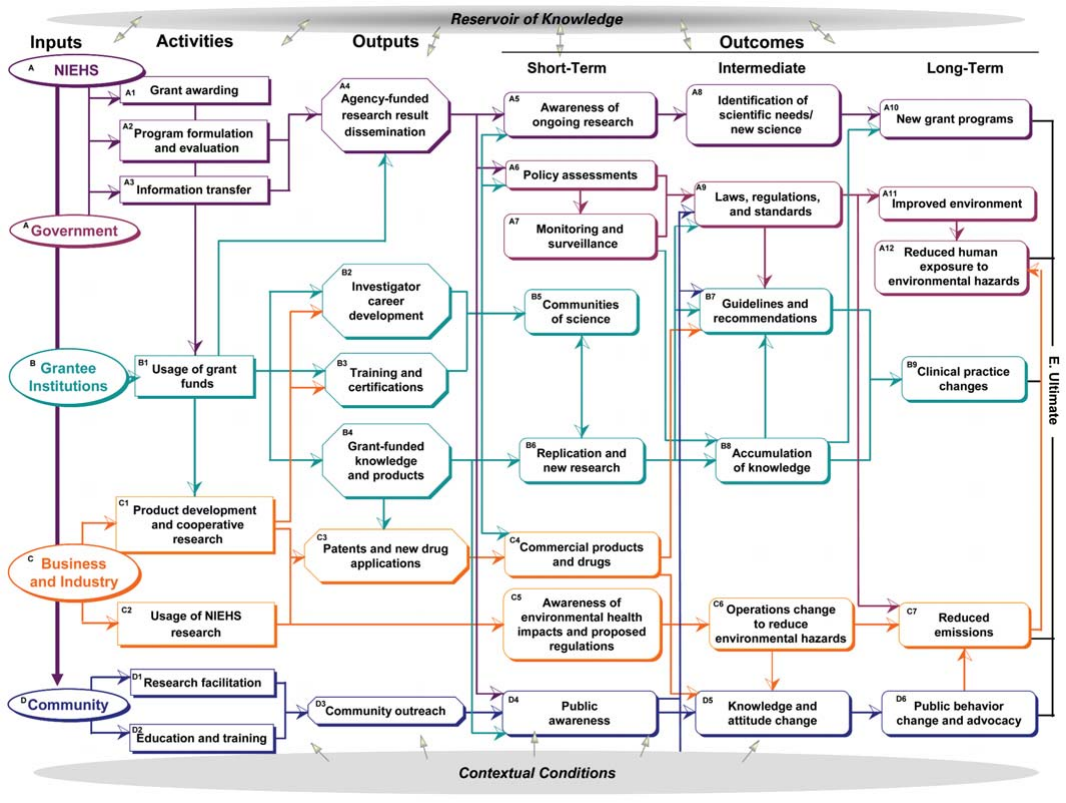
Logic model of the NIEHS extramural research program. Arrows represent linkages between the logic model components. Pathways are identified by letter and color as follows: (*A*) NIEHS and (other) government pathway (purple) illustrates the research process from inputs to outcomes created by, provided by, or carried out by NIEHS as an agency or by NIEHS staff. It also includes the outcomes related to other federal, state, or local government agencies—specifically, policy assessments, monitoring or surveillance systems, and new laws, regulations, and standards—leading to improvements in the environment and resulting in reduced human exposure. (*B*) The grantee institution pathway (light blue) illustrates the research process controlled by grant-funded institutions and conducted by grant-supported investigators. It describes various uses for grant funds for research and development of staff and communities of science, leading to guidelines and scientific knowledge that result in clinical practice changes by health care providers to improved human health. (*C*) The business and industry pathway (orange) illustrates the commercial contribution to the research process provided most frequently by businesses and their representatives, including public–private cooperative research to develop new patents, drugs, products, and services. This institutional path also reflects business and industry’s environmental health impacts; these include their actions and responses to regulations that lead to changes in operations that reduce environmental hazards and reduce emissions. (*D*) The community pathway (dark blue) describes the participation of partners in the research process, such as nongovernmental organizations, community hospitals and clinics, schools, and the general public. The model includes the facilitation of (and participation in) research by the community as well as education and training, community outreach, and public awareness about the research, that in turn results in behavior changes, community advocacy, and personal and local choices that reduce negative impact on human health.

**Figure 2:**
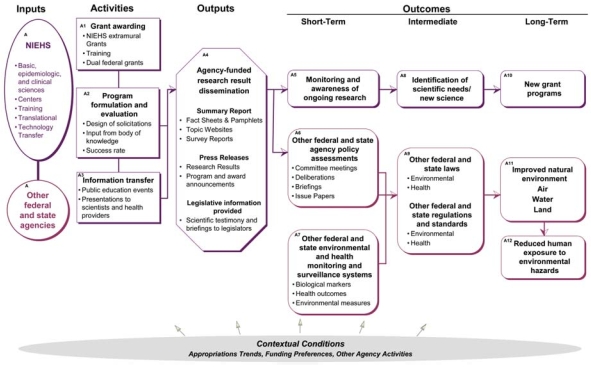
Contribution of NIEHS and other government agencies to the logic model.

**Figure 3:**
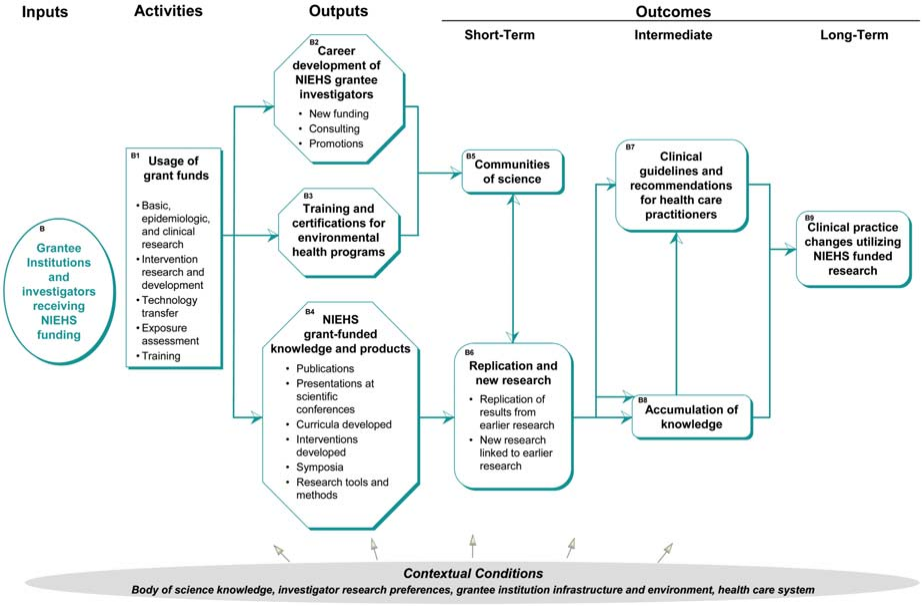
Contribution of grantee institutions to the logic model.

**Figure 4:**
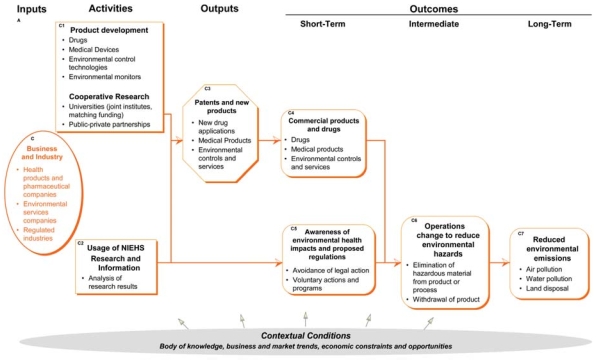
Contribution of business and industry to the logic model.

**Figure 5:**
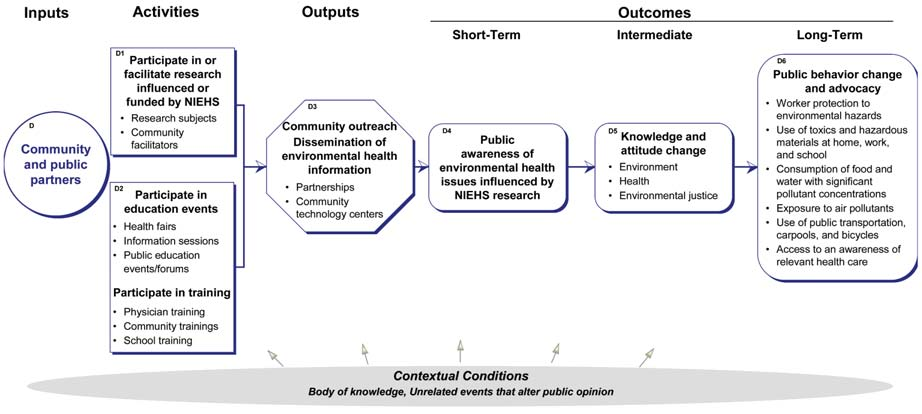
Contribution of community and public partners to the logic model.

The logic model depicted in Figure 1 delineates separate pathways acknowledging contributions by the institutions partnering in the research process: NIEHS, other government (federal, state, and local) agencies, grantee institutions, business and industry, and community partners. Details on these pathways are provided in the submodels depicted in Figures 2–5. Each institutional pathway contains specific logic model elements related to inputs, activities, outputs, and outcomes. Underlying each element are specific metrics (see Table 1 for examples).

Distinctions drawn between the institutional pathways are artificial to some degree, and there is considerable crossover between submodels. Generally, however, each pathway illustrates the research process that would be carried out most directly by a given institutional partner that is being evaluated. This should not be taken to imply that we consider the pathway shown to be the most influential on a particular outcome. In the following sections, we further describe the five institutional pathways and their components. Relationships between the institutions are represented by the arrows connecting components in different institutional pathways. However, relative strength and importance of these relationships cannot be determined from this model.

## Government Pathway: NIEHS and Other Agencies

This pathway describes the inputs, activities, outputs, and outcomes directly associated with the grant programs of both NIEHS and other government agencies (Figure 2, Table 1). Although this is a combined discussion of the two pathways, examples provided relate primarily to NIEHS.

### Inputs

Inputs include funding and resources for NIEHS grant programs and programs of other related agencies such as the U.S. Environmental Protection Agency (EPA), Occupational Safety and Health Administration (OSHA), Food and Drug Administration (FDA), other members of the National Institutes of Health, and the Centers for Disease Control and Prevention. It also includes state and local government agencies that work to improve the environment and human health in their jurisdictions.

### Activities

Activities include those by NIEHS in support of its mission and its extramural grant program, such as research grant awarding to external investigators; information transfer to a variety of audiences such as stakeholder outreach sessions, scientific panels, and information booths; and program formulation of new initiatives. Closely related to these activities is the use of grant funds by grantee institutions (shown in the grantee institution pathway).

### Outputs

Outputs related to the NIEHS and government pathway include summary reports providing a synthesis of scientific information, press releases announcing research results or program activities, and information provided to legislative bodies as policy background. Related outputs are community outreach events conducted by NIEHS and other agencies (shown in the community pathway).

### Outcomes

NIEHS and other government outcomes include those in the short, intermediate, and long term.

- Short term, NIEHS: monitoring and awareness of ongoing research. NIEHS staff maintain an awareness of ongoing environmental health research, whether NIEHS funded or not, to keep abreast of emerging science.
- Short term, government: policy assessments. Before the enactment of new laws and regulations, governmental agencies conduct reviews of research and review recommendations to determine the potential impact of an issue on the environment and human health.
- Short term, government: monitoring and surveillance systems. Monitoring and surveillance measures are put in place by federal, state, or local governments to measure levels of environmental exposures or human disease, sometimes partly in response to reports based on environmental health research. These may be new systems or adaptations of existing systems to measure emerging health hazards.
- Intermediate, NIEHS: identification of scientific needs and new science. Through ongoing monitoring and awareness of environmental health research, NIEHS is able to identify the scientific needs surrounding topics and emerging issues as well as the need for innovative science within the agency’s mission.
- Intermediate, government: laws. Environmental and health-related laws develop from an improved understanding of the relationship between the environment and human health based partly on policy assessments made by NIEHS and other agencies. Major new legislation is relatively rare (compared with regulations) and develops from a combination of awareness of a problem and connection to a policy solution. NIEHS work would most likely contribute to the identification of human environmental health issues.
- Intermediate, government: regulations and standards. Regulatory agencies such as the EPA, FDA, and OSHA (as well as state and local regulatory agencies) promulgate and enforce environmental and health regulations and standards. Regulations and standards published in the Code of Federal Regulations are justified in publicly available staff reports, criteria documents, and technical support documents.
- Long term, NIEHS: new grant programs. Given identification of scientific need and new science, NIEHS formulates new initiatives and programs. This activity is similar to that cited under “Activities” and essentially begins the grant-making process anew, advancing scientific understanding by building on earlier research.
- Long term, government: improved environment. Changes in regulatory standards should improve the natural and built environment. There are many potential measures for intermediate outcomes, so for any particular NIEHS research area, specific physical environmental measures would be selected. In general, these would fall into three broad categories: ambient air pollutant concentrations, lake/river/ocean groundwater quality, and/or land use and soil contamination.
- Long term, government: reduced human exposure to environmental hazards. Reduced human exposure represents decreases in communities’ or citizens’ exposure to environmental hazards that result from regulations and standards. Measures for reduced human exposure are drawn from measures of improvements in the built and natural environments with addition of the number of individuals located within a specified location.

## Grantee Institution Pathway

This pathway describes the inputs, activities, outputs, and outcomes associated with grantee institutions and the research conducted by those institutions (Figure 3).

### Inputs

The inputs describe the staff, financial, and organizational resources of the grantee institution receiving NIEHS funding. The resources are available to the grantee investigators to support the institutions’ research program.

### Activities

These describe the use of the grant funds provided by NIEHS by the grantee institutions. The activities include specific types of research projects that are funded through grants as well as the development of interventions, tools and methods, and other products. Types of activities include basic, epidemiologic, and clinical research; intervention research and development; technology transfer/innovation research; exposure assessments; and training. Related to the activities of universities and other research institutions are the research and development activities of business and industry (shown in the business and industry pathway) and the summary dissemination of results by NIEHS (shown in the NIEHS pathway).

### Outputs

The outputs are the direct products of the grantee institution’s use of NIEHS grant funds. They include tangible products such as presentations, publications, curricula, intervention, and certifications. They also include less tangible products such as knowledge gained from research, new tools and methodologies, and the career development of investigators such as new funding applications, promotions, and membership in committees or working groups that may result from affiliation with NIEHS-funded research. Related to the outputs associated with grantee institutions and investigators is the public awareness of research activities and research results that affect their health and communities (shown in the community pathway), as well as the awareness of NIEHS staff of ongoing research (shown in the NIEHS pathway).

### Outcomes

The grantee outcomes in the model include the following:

- Short term: communities of science. Communities of science are created when investigators working in the same or related areas develop relationships and research networks that contribute to the advancement of knowledge.
- Short term: replication and new research. The use of research findings typically depends upon compilation of evidence from multiple research studies. These can include replication of an initial study and new research that extends earlier studies.
- Intermediate: clinical guidelines and recommendations. Health institutes and professional societies publish clinical guidelines and recommendations related to practice, treatments, and drug use. These are developed based on research and clinical trials that may be funded by NIEHS.
- Intermediate: accumulation of knowledge. Replication and related new research, along with information drawn from other sources, contribute to the accumulation of knowledge and understanding about environmental health. It is the weight of evidence that drives changes in behavior as well as changes in funding priorities.
- Long term: clinical practice changes. As a result of research dissemination, along with the development of laws, policies, and guidelines, health care providers change their practice and treatment behaviors. These changes may be voluntary or regulated, but they are based on the knowledge accumulated through research conducted by NIEHS-funded investigators and others.

## Business and Industry Pathway

This part of the logic model describes the inputs, activities, outputs, and outcomes directly associated with business and industry (Figure 4). It includes research and development activities leading to new commercial products and drugs, as well as the operational and infrastructure changes that industry makes in response to environmental and health hazards.

### Inputs

The inputs describe the major relevant research areas of business and industry that may benefit from NIEHS-funded research, through product development or the use of results to adjust their operations. Industries included are *a*) health care and pharmaceutical companies, *b*) environmental science companies that prevent or reduce pollution and other environmental hazards, and *c*) regulated industries that may produce waste or by-products that are pollutants, or *d*) other environmental hazards.

### Activities

The activities in this submodel include the cooperative research conducted by business and industry with research partners; the development of health and environmental products and services such as drugs, medical devices, and monitors; and the use of research results by business and industry. Cooperative research with universities may contribute to investigator career development (in the grantee institution pathway).

### Outputs

Intellectual property developed by industry is protected by patents. As the result of research and the development of intellectual property, business and industry develop commercial products related to environmental health. These include drugs and medical products to address health issues, and sales of environmental controls and services.

### Outcomes

The business and industry outcomes in the model include the following:

- Short term: commercial products and drugs. The new commercial products developed by business and industry are then sold in the marketplace. Sales represent a short-term outcome because they reflect the amount of influence on health and the environment, as well as measuring benefit to the economy.
- Short term: awareness of environmental health impacts and proposed regulations. Business and industries that are subject to environmental regulations or that produce byproducts that are potential health hazards become aware of the accumulation of research results indicating their potential involvement in environmental health hazards. Outcomes related to this awareness are preregulatory and may include voluntary actions undertaken by companies to avoid legal action or community censure. Although voluntary actions by industry have been found to be limited in reducing emissions compared with mandatory approaches (Morgenstern and Pizer 2007), we include them in the model as potential short-term pathway to change, given the political and administrative constraints to fashioning regulations.
- Intermediate: change in operations to reduce environmental hazards. Specific regulations and sometimes awareness of the environmental impacts of their products and actions encourage businesses and industries to reduce the hazards caused by their operations. These are intermediate outcomes because they are most often in response to laws, standards, and regulations.
- Long term: reduced environmental emissions. As in the government pathway, changes in regulatory standards should improve the natural environment. As the primary polluters or the manufacturers of consumer products that release pollutants, business and industry are the main actors in reducing emissions. There are many potential measures for long-term outcomes, including air pollutant emissions, hazardous waste land disposal, chemical discharges into water bodies, and releases of toxics to all media.

## Community Pathway

This pathway describes the inputs, activities, outputs, and outcomes associated with the community, the general public, that is influenced by or associated with NIEHS extramural funding (Figure 5). The community is also in itself a driver of environmental health impacts in that community activities apart from environmental health research can be a strong influence on broader public policies or research agendas, and promote actions by governmental agencies and business and industry. However, the goal of this model is to show possible mechanisms by which research can influence outcomes, rather than to depict a comprehensive view of how such outcomes may occur.

### Inputs

The inputs describe the staff, financial, and organizational resources of the community and the public partners of NIEHS. In addition to individuals making up the general public, the community includes nongovernmental agencies addressing environmental health or environmental justice, community hospitals and clinics providing health care to the public, and schools.

### Activities

Activities in this pathway are undertaken by the community and public as a result of NIEHS-funded research. The activities include participating in and/or facilitating community-based participatory research; outreach and education such as health fairs, information sessions, and educational forums; and training on environmental hazards to community members or groups such as first responders, teachers, industrial workers, and children/families.

### Outputs

Community outreach including the wide dissemination of environmental health information to the general public, as well as development of public–private partnerships and community technology centers for the advancement of environmental health awareness, is the main output of this pathway.

### Outcomes

The community outcomes in the model include the following:

- Short term: public awareness. Public awareness is an immediate outcome influenced in part by dissemination of NIEHS-funded research through accessible media.
- Intermediate: change in knowledge and attitudes. As a result of awareness raised by NIEHS-funded research, the public’s knowledge and attitudes about the environment, environmental justice, and environmental health issues may be positively influenced.
- Long term: public behavior change and advocacy. Behavior change occurs as a result of changes in knowledge and attitudes about environmental health issues. It includes increased worker protection from environmental hazards; decreased use of toxics and hazardous materials at home, work, and school; decreased consumption of food and water with significant pollutant concentrations; decreased exposure to air pollutants; increased use of public transportation, car pools, and bicycles; and increased access to and awareness of relevant health care. It can also influence business and industry to change practices in response to consumer demand for less toxic and hazardous products.

## Ultimate Outcomes and Contextual Conditions

The connection of research to the ultimate outcomes of improved human health involves multiple steps and actors. Typically, these outcomes would appear 10–50 years after the initial research, as new clinical practices, laws and regulations, and public behavioral changes are implemented and have an effect. The ultimate outcomes are related to the intermediate outcomes of all institutional pathways and fall into two categories: improved human health and well-being and benefit to the economy.

Examples of ultimate outcomes related to improvement of human health include decreases in disease and injuries associated with exposures to adverse environmental health agents. Those associated with benefit to the economy include decreases in health care use, increases in worker productivity, and decreases in worker and school absenteeism due to symptoms and diseases associated with exposures to adverse environmental health agents. Less tangible are increases in value of natural resource goods, services, amenities, and intrinsic value from improved environment.

## Discussion

The value of the logic model lies in its utility in developing pathways by which to link NIEHS-funded research to ultimate outcomes. In addition, metrics associated with each component document the contribution. To illustrate the potential application of the model, we present two brief examples for discussion. These examples demonstrate a simplified approach of how to trace “forward” the influence that research may have on outcomes, even when that influence may be indirect, diffuse, or delayed. This approach does not attempt to identify all of the possible contributing factors to the noted impact.

**Figure 6:**
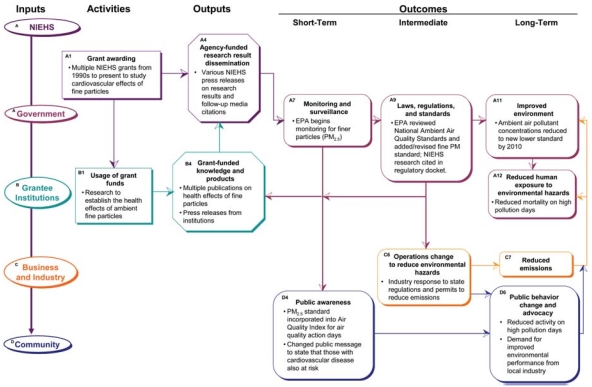
Influence of NIEHS research on cardiovascular disease, fine PM effects, and policy changes. This figure traces how NIEHS-supported research on the role of fine PM in the etiology of cardiovascular disease has led to changes in air quality standards.

Knowledge of the human health effects of ambient airborne pollutants has increased over the last several decades, from an initial focus on ozone and pulmonary diseases such as asthma, to a growing scientific understanding of the effects of fine airborne particulate matter (PM) on cardiovascular disease (e.g., Dockery 2001; Donaldson et al. 2001; Pope et al. 2004). Figure 6 depicts NIEHS-funded research (A1) documenting the health effects of fine PM (B1). For example, NIEHS-funded researchers at Johns Hopkins University published results (B4) of mortality from fine PM in major U.S. cities (Samet et al. 2000) and on hospital admissions related to fine PM (Dominici et al. 2006). Subsequently, research results from these studies and others were disseminated (A4) by NIEHS and the institutions themselves through press releases (e.g., Johns Hopkins University 2000; NIEHS 2006a).

During the last decade, the U.S. EPA shifted its monitoring network to measure finer PM (A7), specifically, PM with diameter less than 2.5 μm (PM_2.5_). The U.S. EPA revised its regulations to include an annual ambient standard for PM_2.5_ (A8), conducting multiple stages of staff and public review of the new standard from the mid-1990s through 2006 (e.g., U.S. EPA 2004). NIEHS-funded research was cited in the regulatory docket (www.regulations.gov) of the later revisions as key evidence for the health effects of PM_2.5_ (e.g., McConnell et al. 1999; Raizenne et al. 1996; Schwartz et al. 1999). States are required to submit implementation plans to achieve compliance with the new ambient standards; as part of these plans, state and local governments pass rules and regulations requiring industry and consumers to change their operations (C6) and reduce emissions (C7). Reduced emissions required by the state implementation plans will improve air quality to the new U.S. EPA standard by 2010 (A11).

In response to research documenting cardiovascular and other health effects, the U.S. EPA added fine PM to its air quality index reporting (U.S. EPA 1999) and specifically included cardiovascular effects in its public health messages (D4) (U.S. EPA 2003). Better knowledge of daily air pollution levels and the fact that those with heart disease are also at risk results in behavior modification by the public to reduce activity during pollution events (Bresnahan et al. 1997) and to advocate to reduce local emissions (D6), thus resulting in reduced human exposure and mortality on high-pollutant days (A12). Multiple studies (including some funded by NIEHS) over the last few decades contributed to and were cited by the EPA when setting and modifying the PM_2.5_ standards.

As the influence is traced through the logic model, it becomes more diffuse and suffers from time discontinuities and lack of documentation. This example illustrates that, with a full evaluation and expert elicitation, it is possible to more specifically identify and semi-quantify the impact of NIEHS research, starting with this overview of potential influence.

**Figure 7:**
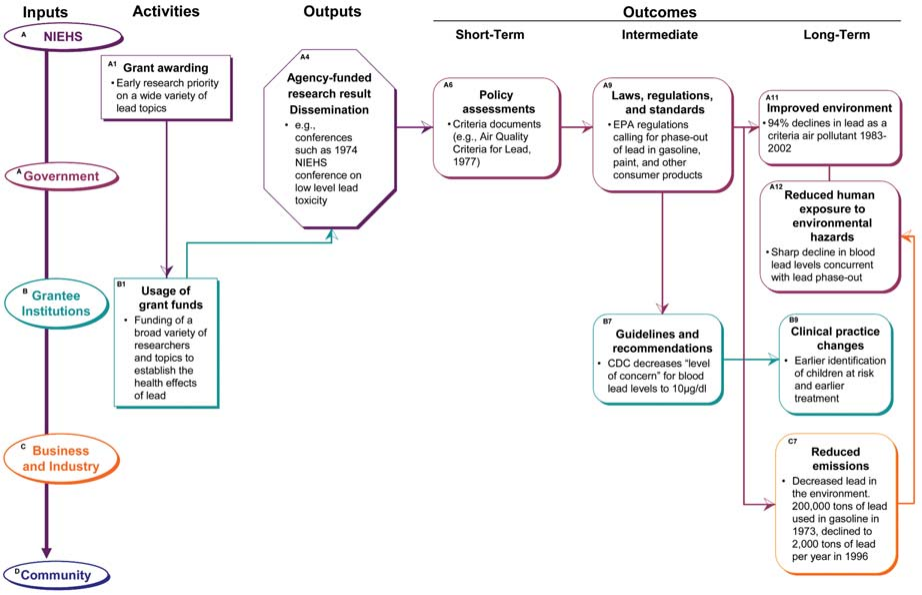
Influence of NIEHS research on a reduction of blood lead levels through policy changes. This figure links NIEHS-supported research to policy changes leading to removal of lead from gasoline and subsequent decreases in human blood lead levels.

The case of lowered blood lead levels through phase-out of leaded gasoline and other lead-containing products demonstrates the influence of a pathway through the logic model related to the impact of government policy changes. Figure 7 shows how activities of grant awarding (A1) and use of grant funds (B1) can lead to dissemination of agency-funded research results (A4). This sets the stage for policy assessment (A6), influencing changes in laws, regulations, and standards changes (A9), which in turn leads to improved environment (A11), reduced emissions from industry (C7), and reduced human exposure (A12), thus leading to ultimate improvements in human health status. The NIEHS has sponsored research on the health effects of lead for more than 20 years (Department of Health and Human Services 2002). Beginning in the 1970s, research funds from federal sources (vs. industry-sponsored studies) were allocated to the study of such health effects particularly in children [reviewed by Needleman (2000)]; NIEHS was a large supporter of these studies. Early studies showed that exposure to low levels of lead during early childhood can lead to delays in cognitive and behavioral development, such as lower IQ levels. Dissemination of these results was accomplished through early conferences and publications on low-lead toxicity; for example, an NIEHS-sponsored conference in 1974, the proceedings of which were published that same year in *Environmental Health Perspectives*. Information from studies like these added to the justification of the need to remove lead from gasoline starting in the 1970s. A criteria document, *Air Quality Criteria for Lead* (U.S. EPA 1977), assessed the scientific basis for regulation, and a standard of 1.5 μg/m^3^ (maximum quarterly calendar average) lead was set in 1978 (U.S. EPA 1978). These air pollution regulations have removed significant amounts of lead from the environment (U.S. EPA 1996, 2002). Data from the second and third National Health and Nutrition Examination Studies show that between 1976 and 1980, there was an average drop in blood lead levels of 30%, concurrent with a 50% reduction in use of leaded gasoline (Annest et al. 1983). These trends have continued with an 86% drop in lead poisoning of children in the United States since the late 1970s and other improvements in health (Meyer et al. 2003; NIEHS 2003). In this example, the NIEHS-funded research on blood lead levels supported ongoing regulations and contributed to the documentation of positive health effects.

The challenge of identifying a specific impact from a research program illustrated in these examples arises from how grant-funded research has an indirect benefit to and little substantial involvement by federal agencies (Federal Grants and Cooperative Agreement Act of 1977). Although fundamental research on both fine PM and blood lead levels contributed to awareness and monitoring of their relevant environmental health issue, the studies were not designed to set standards or to be used in policy decision making, except as an indirect contribution as aggregate knowledge. The 2004 National Research Council report on airborne PM identifies the synthesis of multiple research studies as a requirement for gauging research progress. Although independent research studies may be ideal process for scientific discovery, structured logic models are needed to trace the diffuse yet important role of specific research programs.

## Conclusions

The conceptual logic model for research metrics focuses on NIEHS-funded research programs to measure the contribution of environmental health research to improvements in human health, the environment, and the economy. The model is successfully illustrated here with two brief case examples: effects of PM and blood lead levels. In addition, this logic model approach has been applied to two full case studies—asthma and endocrine disruptors—as part of the larger study, the results of which are to be published separately. Furthermore, a database has been created that maps the logic model components and specific indicators to known published information, online databases, and document repositories that serve as sources of information for measuring outcomes for each logic model component.

Although the main application of the logic model presented here was the environmental health research portfolio of NIEHS, its basic elements are applicable to other environmental or health research programs. The institutions that are part of the research process—government agencies, grantee institutions, business and industry, and community partners—are key players in nearly all environment and health programs. Despite the strengths of this approach, persistent challenges still remain. These include the lack of direct attribution of NIEHS-supported work to many of the outcome measures and the lack of robust electronic databases that can be easily searched to help establish these linkages. Mitigation of these problems will require a stronger effort to include better linkages to the primary literature/grant support and organization of electronic information, particularly policy and/or health guidelines, in an easy format for indexing and searching. This can be achieved only by greater communication among all the stakeholders described in this logic model. We hope that such dialogue will be stimulated by the present study. Finally, this logic model narrows the focus to only one type of input—research—and its potential contribution to impacts. Therefore, it does not attempt to demonstrate all of the many factors that may have contributed to a given impact. It is therefore important for the analyst using this model to not overstate the contribution of research to the impact versus other types of competing influences. The logic model has been developed to apply to diverse programs within NIEHS and will be used as an ongoing program analysis tool. An area of further research is to apply the model to environment and health research programs at other government agencies, universities and research institutions, and private industry.

## Figures and Tables

**Table 1:** Example metrics for logic model components.

ID	Pathway component	Example metrics
NIEHS and other government pathway		
A	NIEHS grant programs	Amount of funding by year, by type
A1	Grant awarding	Number of research grants awarded by year, by type
A2	Program formulation	Amount of funding for new initiatives or programs, by year, by type
A3	Information transfer	Number of staff or grantee testimonies and briefings to decision makers, by year
A4	Agency-funded research result dissemination	Number of press releases (research results, program announcements), by year, by type; number of conferences sponsored by agency, by year
A5	Awareness of research	Number of professional conferences, workshops, and research events attended by NIEHS staff
A6	Policy assessments	Number of policy documents issued that cite NIEHS-funded research, by year
A7	Monitoring and surveillance	Number of monitoring/surveillance measures instituted citing NIEHS-funded research, by year
A8	Identification of scientific needs/new science	Number of new research opportunities identified in NIEHS strategy and planning documents
A9	Laws, regulations, standards	Number of regulations/standards that cite NIEHS-funded research in support documents, by year
A10	New grant programs	Number of new initiatives or programs, by year, by type; amount of funding for new initiatives or programs, by year, by type
A11	Improved environment	Ambient air pollutant concentrations, by year; toxic chemical contamination in indoor environments by location, by year
A12	Reduced human exposure	Pollutant concentrations and measures of exposed populations
Grantee institution pathway			
B1	Use of grant funds	Amount of funding by year, by source; number of investigators/fellows trained under each grant, by year
B2	Investigator career development	Number of grants awarded to investigators by year, by source
B3	Training and certifications	Number and type of certifications provided by investigators by year, by funding source
B4	Grant-funded knowledge/products	Number of presentations at selected key conferences by year, by grant type and funding source; number of peer-reviewed publications by year, by grant type and funding source
B5	Communities of science	Number of NIEHS-funded grants involving interdisciplinary/cross-collegiate principal investigators; number of Memoranda of Understanding between grantee institutions
B6	Replication and new research	Number of citations of previously published research funded by NIEHS, by year (multiple years); impact factor of each citation as measured by ISI
B7	Guidelines/recommendations	Number of clinical guidelines published that cite NIEHS-funded research, by year
B8	Accumulation of knowledge	Number of citations in the literature of previously published research funded by NIEHS, by year
B9	Clinical practice changes	Type of self-reported changes in clinical practice reported by health care providers, by year
Business and industry pathway		
C1	Product development and cooperative research	Amount of industry funding matching NIEHS grant funding, by year; amount of Cooperative Research and Development Agreement (CRADA) funding by year, by type
C2	Use of NIEHS research	Number of industry trade publications that reference NIEHS research
C3	Patents and new drug applications	Number of patents that cite NIEHS-funded research, by year; number of new drugs or products that cite NIEHS-funded research in the patent
C4	Commercial products and drugs	Amount (dollars) from sale of products that cite NIEHS-funded research in the patent or were developed under a NIEHS CRADA, by year
C5	Awareness of environmental health impacts and regulations	Number and source of voluntary programs undertaken by companies that cite NIEHS-funded research as supporting evidence, by year
C6	Operations change to reduce hazards	Number of products or drugs withdrawn from the market, by year; number of businesses that change operations to eliminate hazardous materials, by year
C7	Reduced emissions	Air pollutant emissions inventory, by year; releases of toxics to all media, by year
Community pathway		
D1	Research facilitation	Number of research projects participated in or facilitated in a community, by year
D2	Education and training	Number of persons who receive formal training in a community, by year
D3	Community outreach	Number of outreach events in a community, by year, by type
D4	Public awareness	Number of public awareness campaigns citing NIEHS-funded research, by year
D5	Knowledge/attitude change	Surveys of public’s knowledge and attitude changes regarding key NIEHS issues, by year
D6	Behavior change/advocacy	Surveys of public’s behavior change with regard to key NIEHS issues or topics, by year
E	Ultimate outcomes	Trends in health care use/costs associated with exposures to adverse EH agents, by year; disease-specific mortality rate, by year

ID, identifier.
